# MiR-221, miR-320a, miR133a, and miR-133b as potential biomarkers in leiomyosarcoma

**DOI:** 10.3389/fonc.2025.1577859

**Published:** 2025-06-20

**Authors:** Mst Nasrin Akhtar, Annabell Walter, Kathrin Katenkamp, Yuan Chen, Thomas Lehmann, Wolfram Weschenfelder, Christian Spiegel, Matthias Vogt, Gunther O. Hofmann, Andreas Hochhaus, Nikolaus Gaßler, Joachim H. Clement, Karin G. Schrenk

**Affiliations:** ^1^ Abteilung für Hämatologie und Internistische Onkologie, Klinik für Innere Medizin II, Universitätsklinikum Jena, Jena, Germany; ^2^ Mitteldeutsches Krebszentrum, Standort Jena, Jena, Germany; ^3^ Institut für Rechtsmedizin, Sektion Pathologie, Universitätsklinikum Jena, Jena, Germany; ^4^ Institut für Medizinische Statistik, Informatik und Datenwissenschaften, Universitätsklinikum Jena, Jena, Germany; ^5^ Klinik für Unfall-, Hand- und Wiederherstellungschirurgie, Universitätsklinikum Jena, Jena, Germany; ^6^ Klinik im Medizentrum PartGmbB, Erlangen, Germany

**Keywords:** miRNA, miR-221, miR-320a, miR-133a, miR-133b, sarcoma, biomarkers

## Abstract

**Background:**

Leiomyosarcoma is an aggressive tumor with a high rate of distant metastasis and poor prognosis. No standardized biomarkers are available to assess early diagnosis or monitoring during the clinical course. MicroRNAs (miRNAs) function in modulating a multitude of targets and are involved in tumorigenesis, cancer progression, and metastasis. This study was designed to evaluate miR-221, miR-320a, miR-133a, and miR-133b as potential biomarkers in leiomyosarcoma.

**Materials and methods:**

The expression levels of miR-221, miR-320a, miR-133a, and miR-133b as well as their target mRNAs *CDKN1B*, *TGFBR1*, and *IGF1R* were assessed by quantitative real-time reverse transcription polymerase chain reaction (qRT-PCR) in tissue samples from 33 patients with leiomyosarcoma. Wilcoxon test, Kruskal-Wallis test, Mann-Whitney test as well as Spearman-Rho-test were used for statistical analysis. Receiver operating characteristic (ROC) analyses were performed to discriminate metastatic risk of local and primary tumors in correlation to miR-221, miR-320a, miR-133a, and miR-133b.

**Results and discussion:**

The expression levels of miR-221, miR-320a, and miR-133a were significantly upregulated in leiomyosarcoma tumor tissue compared to adjacent non-tumor tissue (p = 0.003 for miR-221, p = 0.006 for miR-320a, and p = 0.044 for miR-133a respectively). The target mRNAs *CDKN1B*, *TGFBR1*, and *IGF1R* in 25 leiomyosarcoma tumor tissues were not significantly deregulated. There was no significant upregulation in primary tumors and metastases compared to local tumors for miR-221, miR-320a, miR-133a, and miR-133b. ROC curves of miRNA-221, miR-320a, miR-133a, and miR-133b to predict metastatic risk at initial presentation of the tumor, comparing non-metastasizing and metastasizing leiomyosarcomas, demonstrated no significant levels.

**Conclusion:**

miR-221, miR-320a, and miR-133a were significantly upregulated in leiomyosarcoma tumor tissue as compared to adjacent non-tumor tissue. There was no significant difference in miRNA expression and ROC curves in primary tumors as compared to local tumors. While not statistically significant, ROC curve of miR-133b suggests a potential role in predicting metastatic risk, warranting subsequent analysis. This study provides evidence for further evaluation of miR-221, miR-320a, miR-133a, and miR-133b as biomarkers in primary diagnosis and assessment of metastatic risk in leiomyosarcoma.

## 1 Introduction

Sarcomas are a heterogeneous group of neoplasms arising from mesenchymal tissues such as bone, cartilage, muscle, and other connective tissues. The discovery of more than 150 distinct sarcoma subtypes differing in pathophysiology, clinical presentation, genetic features, and therapeutic response highlights their heterogeneity. Sarcomas are very rare tumors with an incidence of soft tissue sarcomas of 6/100–000 cases/year in Germany. Approximately 11% of all soft tissue sarcomas are leiomyosarcomas (LMS) derived from smooth muscle with a high risk of distant metastasis, leading to dismal outcomes with a 5-year survival rate of 35% (1–3). Due to their rarity, many studies in sarcomas are performed by summarizing several entities, leading to heterogenous results. For this reason inclusion of one sarcoma entity into analyses is mandatory with the caveat of low patient numbers. Molecular analyses in LMS demonstrated complex karyotypes and alterations in TP53 in 92% of LMS cases, RB1 in 94%, PTEN in 86%, and a high rate of mutations in DNA damage response pathways, leading to genomic instability (1, 4, 5). To improve diagnosis and prognosis, identification of molecular biomarkers is warranted. In recent years, several molecular biomarker candidates have been identified in leiomyosarcoma, including the insulin-like growth factor 1 receptor (IGF1R), MDM2, TP53, fragile histidine triad (FHIT), and microRNAs (6), however, a clinical evaluation of these biomarkers was not performed.

MicroRNAs (miRNAs) are small noncoding RNAs, transcribed from nonprotein coding genes or introns that modulate gene expression, mainly through translational inhibition or degradation of messenger RNAs (mRNAs) (7, 8). MicroRNAs are single-stranded RNAs consisting of ~22 nucleotides that regulate protein expression by pairing with the 3´untranslated region (3´UTR) of target mRNAs (8). The 5–8 nucleotides seed sequence at the 5´end of the miRNA binds to complementary sequences in the 3´untranslated regions (3´UTRs) of the target mRNA, mediating translational repression or transcriptional degradation of the target mRNA (9, 10). Certain miRNAs might possibly bind to the 5´untranslated region (5’ UTR) and the open reading frame (ORF) region, however, in such cases they occur less frequently and work less effectively (11).

One key aspect of miRNA biology is that a single miRNA can target hundreds of mRNAs, controlling entire signaling networks. On the other hand, several miRNAs may target a single mRNA (12). Nearly all biological activities depend on miRNAs including cell growth, proliferation, and differentiation as well as metabolism and development (13). Dysregulation of miRNA expression is associated with many human diseases, including cancer (14). Consequently, a growing number of studies have shown that, depending on the cellular context and the target genes, miRNAs can act as potential oncogenes (oncomiRs) or oncosuppressor genes (oncosuppressor-miRs) (15).

Multiple array analyses have been performed to identify characteristic miRNA cancer signatures. To use these results in the clinical setting is a major challenge at the present time. MiRNAs involved in tumorigenesis of leiomyosarcoma are miR-221, miR-320a, miR-133a, and miR133b (16–18). MiRNA-221 is encoded in tandem from a gene cluster located in chromosome Xp11.3 (19). MiR-221 expression is up-regulated in several human malignancies, suggesting that it has an oncogenic function in the development and spread of cancer (20–22). Overexpression of miR-221 results in reduced expression of the cell cycle inhibitor P27kip1. *CDKN1B* mRNA was identified as a target of miR-221 (23). The *CDKN1B* gene encodes for the cyclin-dependent kinase (CDK) inhibitor P27/kip1 that acts to suppress cell cycle progression (24) from G1 to S phase by binding to the CDK2 and cyclin E complex (25). High P27kip1 protein expression has been described in quiescent cells and its loss is a common characteristic in carcinomas, occurring even before invasion, as evidenced by both carcinoma *in situ* and invasive tumor components (26). Alteration of miR-320a expression has been implicated in multiple cancers as well. MiR-320a functions both as a tumor suppressor and an oncogene (27–30) and targets *TGFBR1* (31). The miR-133 family (miR-133a, miR-133b) is involved in the development of skeletal and cardiac muscle (32, 33). In several cancers miR-133a was described as a tumor suppressor (34–41). In addition, miR-133b expression is downregulated in several cancers, suggesting a crucial role for miR-133b in carcinogenesis and cancer progression. (42–46). By targeting the insulin-like growth factor 1 receptor (*IGF1R*) and inhibiting the downstream AKT and ERK signal pathway, miR-133a limits cell proliferation, causes cell cycle arrest at the G0/G1 stage, and increases cell apoptosis (47, 48). Increased expression and activity of IGF1R have been documented in multiple forms of tumors, and contributes to the enhancement of cancer cell growth and evasion of programmed cell death (49, 50). The specific functional significance of the dysregulation in LMS of the target mRNAs *CDKN1B, TGFBR1, IGF1R* has not been evaluated. We therefore included expression analyses of these target mRNAs into this study.

We sought to investigate the potential of miR-221, miR-320a, miR-133a, and miR-133b, along with their target mRNAs *CDKN1B*, *TGFBR1*, and *IGF1R*, as candidate biomarkers of leiomyosarcoma. This research involves a series of experimental studies to validate the utility of these biomarkers in LMS patients. It includes profiling the expression of miRNAs-221, miR-320a, miR-133a, and miR-133b in LMS tumor tissue and corresponding adjacent non-tumor tissue, analyzing their target mRNAs, and comparing miRNA expression in local tumors, primary tumors and metastases. Moreover we analyzed the diagnostic ability of miRNAs to predict metastasis at initial presentation of LMS and correlated miRNA expression with clinical and histopathological information to identify miRNAs as potential biomarkers in leiomyosarcomas.

## 2 Materials and methods

### 2.1 Patients and samples

33 patients were included into this study. The Ethics Committee of the Friedrich-Schiller University Jena approved this study with approval number: Reg.-Nr.: 2022-2661_1-Material. Written informed consent was obtained from 28 patients or their relatives. Five patients included into this study had passed away and no family contacts were available. According to the recommendations of the Central Ethics Committee Germany of the Bundesärztekammer, the use of such samples is possible under defined conditions. All requirements were met and approved by the local Ethics Committee of the Friedrich-Schiller University Jena. A total of 33 leiomyosarcoma specimens and corresponding non-tumor tissues were collected from the Institute of Forensic Medicine, Section Pathology, University Hospital Jena. Formalin-fixed paraffin-embedded (FFPE), non-necrotic tissues were employed for the analysis. Clinical characteristics of the patients were age, gender, disease stage, grade, time of first presentation, affected organ, and time to metastasis (**Supplementary Table 1**). For local and primary tumors tissue samples were obtained at the time of initial presentation before start of treatment. Material from metastases was obtained at different time points when it was clinically indicated. Local tumors were defined as no metastases at initial presentation or later during the clinical course. Primary tumors showed presence of metastases either at initial presentation or later on progression.

### 2.2 RNA isolation

The extraction of total RNAs, including miRNAs, from formalin-fixed paraffin-embedded (FFPE) samples, was carried out using the miRNeasy FFPE-Kit (QIAGEN, Hilden, Germany) following the manufacturer’s protocol. The starting material for RNA purification were freshly cut sections of FFPE tissue. The first step involved deparaffinization of the tissue sections and extraction of total RNA from microdissected tissues. Deparaffinization was achieved primarily through immersion in 100% xylene and drying at room temperature (RT). The slides were then washed with 100% ethanol to remove any remaining paraffin. The embedded tissue sections were carefully removed from the slides using a sterile blade or scalpel and transferred into nuclease-free tubes. Subsequently, total RNA extraction was performed following the instructions in the miRNA easy FFPE kit manual. The quantity of the total RNAs were determined using a Nanodrop 2000 spectrophotometer. RNA purity and integrity was assessed by measuring A260/A280 absorption and classical PCR followed by gel electrophoresis. Clear bands were observed for each miRNA target, indicating that the RNA quality was sufficient. The isolated RNA was either stored at -80°C for future use or directly used for reverse transcription.

### 2.3 cDNA synthesis

cDNA for miRNA (hsa-miR-221-5p, hsa-miR-320a-5p, hsa-miR-133a-5p, and hsa-miR-133b-5p) was synthesized from FFPE tissue specimens by using All-in-one™ kit (Gene Copoeia™, Rockville, MA, USA). Firstly, 1 µl of total RNA was employed as starting material. 1 µl of 2 U/µl polymerase and 1 µl of SureScript™RTase mix (20x) were added. Additionally, 4 µl of 5 x PAPRT buffer was introduced to the mixture. The final volume was adjusted to 20 µl using ddH_2_O. The mixture was then incubated at 37°C for 60 min, following a brief centrifugation step. After the incubation step, the reaction mixture was kept at 85°C for 5 min. The cDNA synthesis process was performed using a TRIO thermocycler (Biometra, Göttingen, Germany). The resulting cDNA was either used immediately or stored at -20°C until future use.

The process of transcribing mRNA into cDNA was carried out by generating cDNA from RNA obtained from cell cultures and tissue specimens using the TRIO thermocycler (Biometra). Initially, 1 µg of RNA was diluted in 8.5 µl RNase-free water and denatured at 65°C for 5 min. Next, 11.5 µl of reverse transcription mix was added to each sample, resulting in a final volume of 20 µl and incubated as follows: RT for 10 min, 37°C for 60 min, and 95°C for 5 min. The synthesized cDNA was either used immediately or stored at –20°C until further use.

### 2.4 MiRNA expression profiling by quantitative polymerase chain reaction

The expression levels of hsa-miR-221-5p, hsa-miR-320a-5p, hsa-miR-133a-5p, and hsa-miR133b-5p were measured by using All-in-one™ miRNA qRT-PCR detection kit 2.0 (BioCat, Heidelberg, Germany). *SNORD49A* and *RNU6–2* were selected as references for normalizing the expression levels in the tissue samples. All-in-one miRNA qPCR primer (BioCat) was used as forward primer and universal adapter primer was used as a reverse primer. The reverse transcription reaction product was diluted 25 times before being used for quantification using qRT-PCR. In the qRT-PCR reaction, 8 µl of the master mix and 2 µl of the diluted cDNA were used to obtain a final reaction volume of 10 µl. The rotor-q-gene (QIAGEN) system was used to conduct the qRT-PCR experiments. The qRT-PCR program using the rotor-q-gene system included an initial denaturation at 95°C for 10 min and 40 cycles of denaturation (95°C for 10 sec), annealing (61°C for 20 sec), and extension (72°C for 10 sec). Each reaction had a positive control, which was a sample of miRNA from a cell line, and a negative control, which was a reaction without any cDNA. The mean cycle threshold (Ct) value was obtained from the two duplicates of each PCR reaction using the rotor-q-gene system. Further analysis of the qRT-PCR results was performed using mean Ct values.

### 2.5 Cell culture

#### 2.5.1 Cell lines used as positive controls

The cell lines used in this study were as follows: FaDu (ATCC^®^HTB-43™), HCC78(DSMZ, ACC563), SkBr-3 (ATCC^®^HTB-30™), HepG2 (DSMZ, ACC180), and normal lung tissue. Cell lines were used to identify positive controls and to establish an optimal quantification procedure for each miRNA to enable comparison of expression levels, further enhancing the validity and reproducibility of our findings.

In FaDu cell line miR-320a and miR-133a expression was found. MiR-320a and miR-133b were detected in a lung tissue sample (#6190). Positive expression of miR-320a and miR-133a was observed in HCC78, while negative expression was observed for miR-221 and miR-133b. HepG2 tested positively for the expression of miR-320a and miR-221, but negatively for the expression of miR133a and miR-133b. In SKBr-3 expression of miR-320a and miR-133b was positive (**Supplementary Table 2**).

#### 2.5.2 Cell cultivation

HCC78, FaDu, SkBr-3, and HepG2, were cultured in flasks using RPMI 1640+ GlutaMAX™-I medium. The medium was further supplemented with either 10% (HCC78, FaDu, SkBr-3) or 20% (HepG2) fetal calf serum (FCS) depending on the cell line. The flasks were then kept in a humidified incubator at 37°C and 5% CO_2_.

The cells were washed with Dulbecco’s phosphate buffer saline (D-PBS) once they had reached 90% confluence. They were removed using 0.05% Trypsin-EDTA from the cell culture flask’s bottom. The cells were then split into ratios ranging from 1:3 to 1:5 and grown in fresh RPMI1640+ GlutaMAXTM-I media that was supplemented with 10% or 20% FCS.

### 2.6 Polymerase chain reaction

#### 2.6.1 Qualitative polymerase chain reaction assay for mRNA

This study employed a two-step PCR approach to amplify and quantify the desired targets. Initially, a qualitative PCR was performed with a limited number of cycles to increase the concentration and specificity of the cDNA of the targets. This was followed by a quantitative PCR using the same primers, which allowed for accurate quantification of the targets. The mRNA expression level of the targets *CDKN1B, TGFBR1, IGF1R* and the internal control *RPL37A* were measured using the qRT-PCR method. The following primer sequences were used for the amplification of specific target genes, along with their respective expected product lengths (**Supplementary Table 3**). PCR was performed by adding 22.5 µl of the PCR master mix to 1 µl of cDNA produced from reverse transcription. This conventional PCR was a 10-cycle PCR and different programs were used for each target.

#### 2.6.2 Quantitative analysis of mRNA expression levels

The PCR product from conventional PCR was used for quantification. 1 µl of each resulting product was used as template in the second qPCR amplification using rotor-q-gene by SYBR^®^ Green detection chemistry. Briefly, qPCR amplification was performed in a 10 µl final reaction volume containing 500 nmol/L of each primer used in the first RT-PCR reaction and 1×SYBR^®^ Green PCR Master Mix. The initial concentration of *CDKN1B*, *TGFBR1*, *IGF1R*, and the internal control *RPL37A* mRNA were assessed using the above described RT-PCR products as standard templates for further amplification with the same primers. The target mRNA was standardized to *RPL37A* mRNA expression. qRT-PCR was performed to analyze the expression levels of three different target mRNAs, *CDKN1B*, *TGFBR1*, and *IGF1R*. The qRT-PCR protocol involved an initial denaturation step at 95°C for 3 min, followed by a varying number of cycles of denaturation, annealing, and extension. For *TGFBR1*, the qRT-PCR program involved a total of 34 cycles of denaturation at 95°C for 20 sec, annealing at 61°C for 20 sec, and extension at 72°C for 20 sec. For *IGF1R*, the qRT-PCR program involved an initial denaturation step at 95°C for 3 min, followed by 34 cycles of denaturation at 95°C for 30 sec, annealing at 60°C for 30 sec, and extension at 72°C for 30 sec. qRT-PCR for *CDKN1B* included initial denaturation at 95°C for 3 min, followed by 34 cycles of denaturation at 95°C for 30 sec, annealing at 60°C for 30 sec, and extension at 72°C for 30 sec. All qRT-PCR experiments were performed in duplicates using a real-time PCR system, and the expression levels were calculated using the comparative Ct (threshold cycle) method.

### 2.7 Statistical analysis

The expression of miRNAs was measured using Ct values. The cycle threshold (Ct) is defined as the number of cycles necessary for the fluorescent signal to cross the threshold in qRT-PCR. Ct values of miRNA were normalized to the references small nuclear RNA *U6 (RNU6-2)*, and *SNORD49A* to obtain ΔCt-values. Normalization of the target mRNA Ct values was performed with *RPL37A* mRNA. ΔCt was calculated as ΔCt = Ct (miRNA or mRNA) − Ct (reference) and ΔΔCt as ΔΔCt = ΔCt (tumor sample) − ΔCt (non-tumor sample). The relative expression of miRNAs and target mRNAs was obtained by using the 2^−ΔΔCt^ method, where ΔΔCt represents the log 2-fold change and the fold change was calculated as 2^−ΔΔCt^. Times of regulation was determined as 1/fold change. Results are presented as median ± interquartile range (IQR). Data were analyzed by SPSSv29.0 (IBM, Ehningen, Germany). Wilcoxon test was used for calculating the statistical significance of observed expression differences between groups, p < 0.05 was considered significant. Comparison of expression levels between local tumors, primary tumors, and metastases was determined with the Mann-Whitney test. The evaluation of miRNAs as biomarkers for prediction of metastasis at initial presentation was performed by receiver operating characteristic (ROC) curves and area under curve (AUC) with 95% confidence interval. For correlation of clinical and histopathological findings with miRNA expression the Spearman-Rho test was used. All analyses were exploratory and no correction for multiple testing was performed.

## 3 Results

### 3.1 Evaluation of miR-221, miR-320a, miR-133a, miR-133b and their target mRNAs *CDKN1B*, *TGFBR1*, and *IGF1R* in leiomyosarcoma tissue

The expression levels of miRNAs in tumor tissues and adjacent non-tumor tissues obtained from 33 patients with LMS were examined by qRT-PCR. By normalization to the small nuclear RNA *U6 (RNU6-2)* and *SNORD49A* ΔCt values were obtained. 2^-ΔΔCt^ method was used to calculate fold change. For comparison of miRNA expression between tumor and adjacent non-tumor tissue Wilcoxon test was performed and p < 0.05 was considered significant.

**Figure 1:**
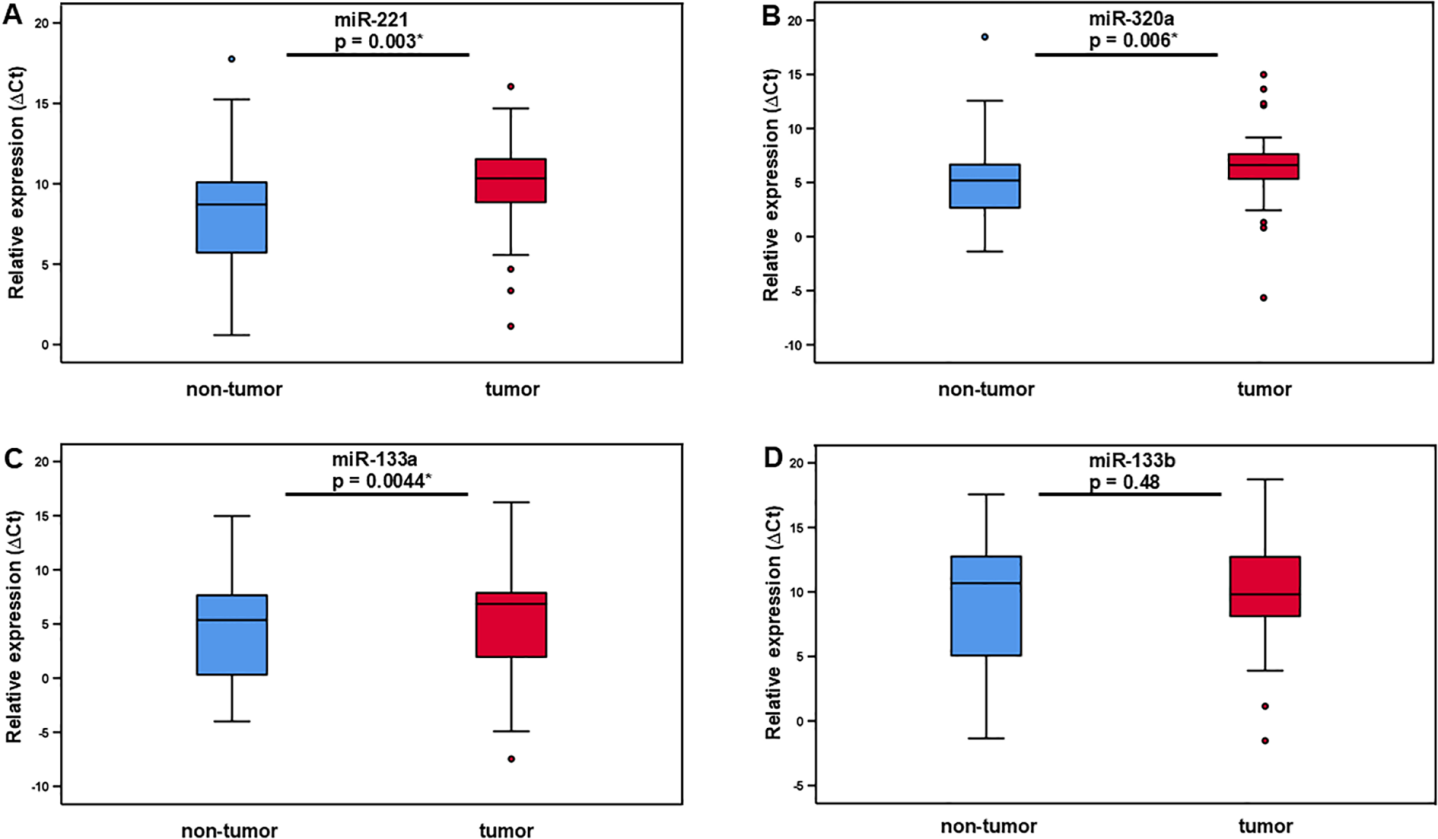
Relative expression of miR-221, miR-320a, miR-133a, and miR-133b in LMS tumor tissue and adjacent non-tumor tissue. The level of miR-221 **(A)**, miR-320 **(B)**, and miR-133a **(C)** expression is significantly higher in tumor tissue compared to adjacent non-tumor tissue. MiR-133b expression in tumor compared non-tumor tissue showed no significant difference **(D)**.

As shown in **Figure 1** and **Table 1** miR-221, miR-320a, and miR-133a median ΔCt values of expression levels were higher in LMS samples compared to adjacent non-tumor tissue. MiR-221 expression levels had a median ΔCt value of 10.34 (IQR ±3.1) while non-tumor tissue had a median ΔCt value of 8.71 (IQR ±4.47). Using the 2^-ΔΔCt^ method the median fold change was 0,36 (IQR ±0.98), indicating a 2.8 times upregulation of miR-221 in tumor tissue compared to non-tumor tissue. Our analysis using the Wilcoxon test revealed a statistically significant difference (p = 0.003) in miRNA-221 expression levels between tumor tissue and non-tumor tissue (**Figure 1A**, **Table 1**). The expression level of miR-320a in tumor tissue had a median ΔCt value of 6.61 (IQR ±2.54), in non-tumor tissue a median ΔCt value of 5.19 (IQR ±4.11), with a median fold change of 0.40 (IQR ±1.02), and a 2.5 times upregulation. The difference in miR-320a expression levels between tumor and non-tumor tissue is statistically significant (p = 0.006, Wilcoxon test, **Figure 1B**, **Table 1**). MiR-133a expression median ΔCt value was 6.85 (IQR ±6.33) in tumor tissue, in non-tumor-tissue 5.36 (IQR ±7.48) with a median fold change of 0.5 (IQR ±1.27), a 2.0 times upregulation and a significance of p = 0.044 in the Wilcoxon test (**Figure 1C**, **Table 1**). MiR-133b expression levels in tumor tissue exhibited a ΔCt median value lower compared to non-tumor tissue. MiR-133b median values in tumor tissue was 9.81 (IQR ±5.83), in non-tumor tissue 10.68 (IQR ±8.22), fold change 1.27 (IQR ±2.18), downregulation 0.79 times (p = 0.48, **Figure 1D**, **Table 1**).

**Table 1:** Fold change and p-values of miR-221, miR-320a, miR-133a, miR-133b and their target mRNAs *CDKN1B*, *TGFBR1*, *IGF1R*.

N = 33				
miRNA	miR-221	miR-320a	miR-133a	miR-133b
Fold change	0.36	0.4	0.5	1.27
IQR (±)	0.98	1.02	1.27	2.18
p-value (ΔCt)	0.003*	0.006*	0.044*	0.48
N = 25				
target	*CDKN1B*	*TGFBR1*	*IGF1R*	
Fold change	1.83	1.42	0.96	
IQR (±)	10.5	9.68	9.25	
p-value (ΔCt)	0.056	0.126	0.148	

*significant p < 0.05.

**Figure 2:**
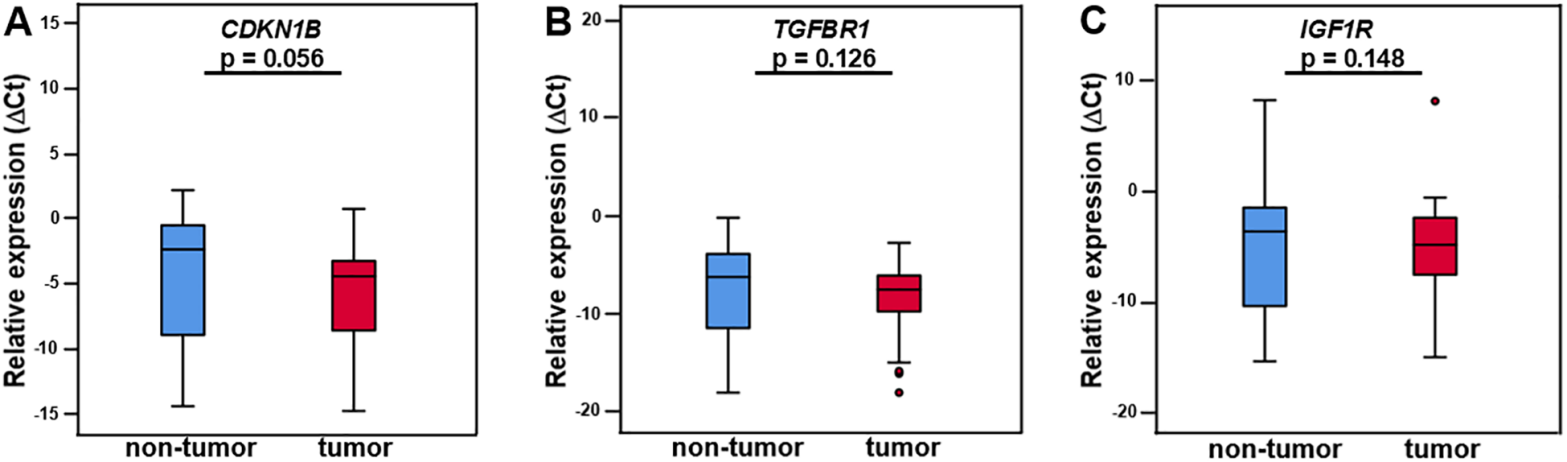
*CDKN1B*, *TGFBR1*, and *IGF1R* mRNA in LMS tumor tissue and adjacent non-tumor tissue. The differential expression of *CDKN1B, TGFBR1*, and *IGF1R* did not differ significantly between tumor and non-tumor tissue **(A–C)**. N = 25. The expression levels are examined by real-time qPCR and normalized to the reference genes *SNORD49A* and *RNU6–2* to obtain ΔCt values. The presented data are the median ΔCt values +/- IQR (interquartile range) and p-values. p < 0.05 is considered statistically significant (*).

Expression difference of the miRNA-221 target mRNA *CDKN1B* in tumor tissue compared to adjacent non-tumor tissue was not significant, with a *CDKN1B* tumor tissue median ΔCt value of -4.45 (IQR ±5.65), non-tumor tissue median ΔCt value of -2.39 (IQR ±8.46), fold change 1.83 (IQR ±10.5), 0.54 times downregulation and p-value of 0.056 (Wilcoxon test, **Figure 2A**, **Table 1**). There was no discernible difference between the miR-320a target mRNA *TGFBR1* in tumor tissue and the nearby non-tumor tissue. The median ΔCt value of *TGFBR1* in tumor tissue was -7.5 (IQR ±4.45), in non-tumor tissue -6.25 (IQR ±8.23), fold change 1.42 (IQR ±9,68), 0.70 times downregulation and p = 0.126 (**Figure 2B**, **Table 1**). The expression of the miR-133a and miR-133b target mRNA *IGF1R* in tumor tissue did not differ significantly from the neighboring non-tumor tissue. *IGF1R* in tumor tissue had a median ΔCt value of -4.92 (IQR ±5.43), in non-tumor tissue -3.71 (IQR ±9.28), fold change 0.96 (IQR ±9.25) and p-value of 0.148 (**Figure 2C**, **Table 1**).

### 3.2 Stage-specific profiling of miRNAs in leiomyosarcoma comparing miR-221, miR-320a, miR-133a, and miR-133b expression in local, primary, and metastatic tumor tissue

**Figure 3:**
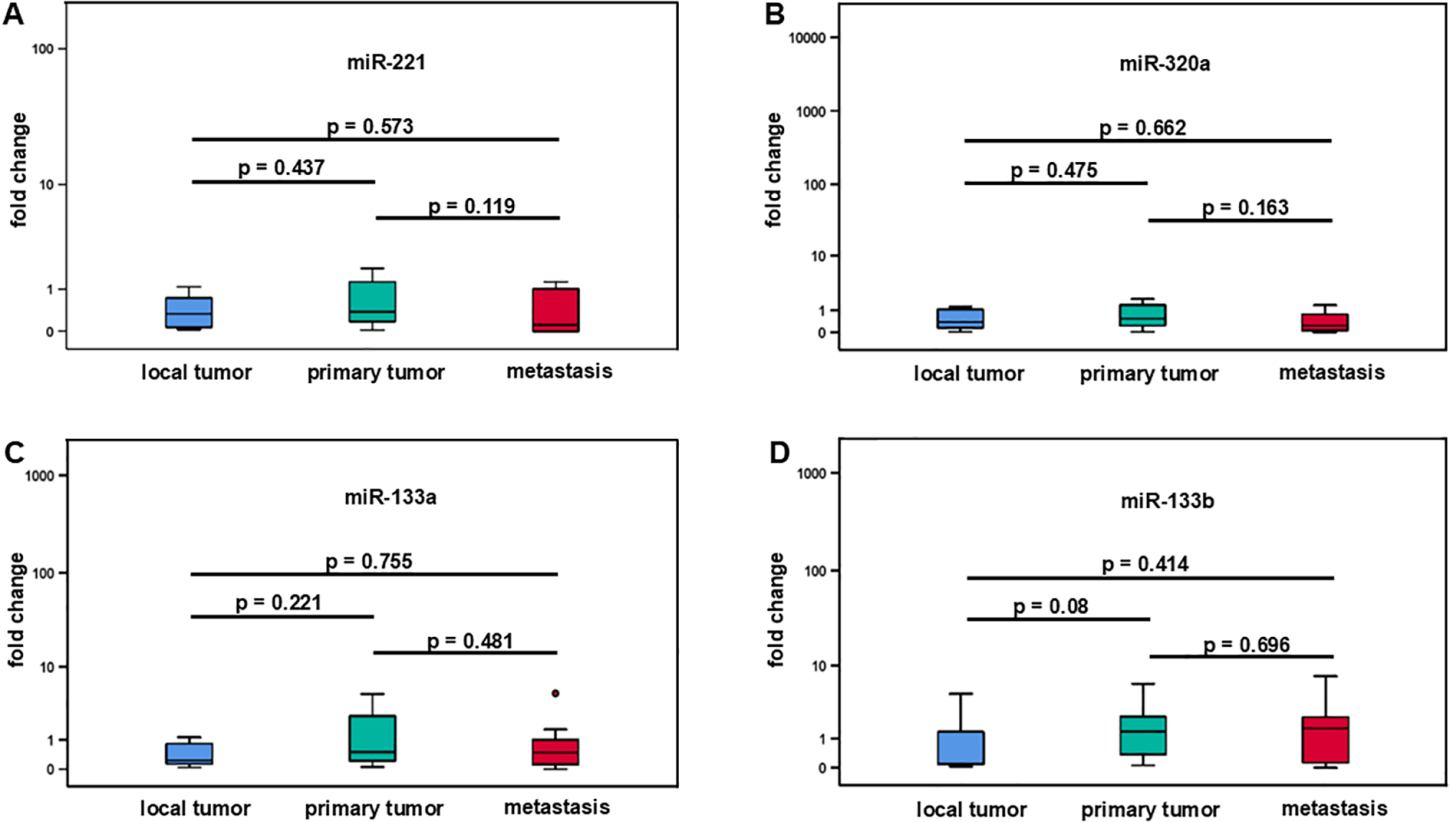
Comparative analysis of miR-221, miR-320a, miR-133a, and miR-133b expression in different stages of leiomyosarcoma. Local tumors (N = 6), primary tumors (N = 19), and metastases (N = 8). No significant difference between local tumors and primary tumors, local tumors and metastases as well as between local tumors and primary tumors was demonstrated for miR-221, miR-320a, miR-133a, and miR-133b **(A-D)**.

In this analysis, we compared the change of expression levels of miR-221, miR-320a, miR-133a, and miR1-33b in local, primary, and metastatic tumor tissue of patients with LMS. Local tumors did not metastasize (N = 6), while primary tumors were metastasized at the time of tumor detection or later during the clinical course (N = 19). Tissue from metastases was obtained from 8 patients. Using the Mann-Whitney test, there was no significant change in miR-221 expression between local tumors and primary tumors with a median fold change of 0.33 (IQR ±0.75) versus 0.38 (IQR ±1.25), and p = 0.437 (**Figure 3A**, **Table 2**). Similarly local tumors compared to metastases revealed no significant difference in miR-221 expression with a median fold change of 0.33 (IQR ±0.75) versus 0.12 (IQR ±1.09), and p = 0.573. There was no significant difference in miR-221 expression between primary tumors and metastases with a fold change of 0.38 (IQR ±1.25) versus 0.12 (IQR ±1.09), and p = 0.119 (**Figure 3A**, **Table 2**). MiR-320a, miR-133a, and miR-133b expression showed no significant change between local and primary tumors, local tumors and metastases or local tumors and primary tumors respectively (**Figures 3B–D**, **Table 2**).

**Table 2:** Fold change and p-values of miR-221, miR-320a, miR-133a, and miR-133b comparing local tumors, primary tumors, and metastasis.

miRNA	miR-221	miR-320a	miR-133a	miR-133b
local tumor (fold change)	0.33	0.38	0.23	0.11
local tumor (IQR ±)	0.75	0.97	0.78	2.1
primary tumor (fold change)	0.38	0.54	0.5	1.34
primary tumor (IQR ±)	1.25	1.21	3.04	2.14
metastasis (fold change)	0.12	0.24	0.48	1.54
metastasis (IQR ±)	1.09	0.93	1.21	2.46
local tumor/primary tumor (p)	0.437	0.475	0.221	0.08
local tumor/metastasis (p)	0.573	0.662	0.755	0.414
primary tumor/metastasis (p)	0.119	0.163	0.481	0.696

*significant p < 0.05.

Total N = 33; local tumor N = 6; primary tumor N = 19; metastasis N = 8.

### 3.3 Diagnostic accuracy of miR-221, miR-320a, miR-133a, and miR-133b for prediction of metastatic risk

**Figure 4:**
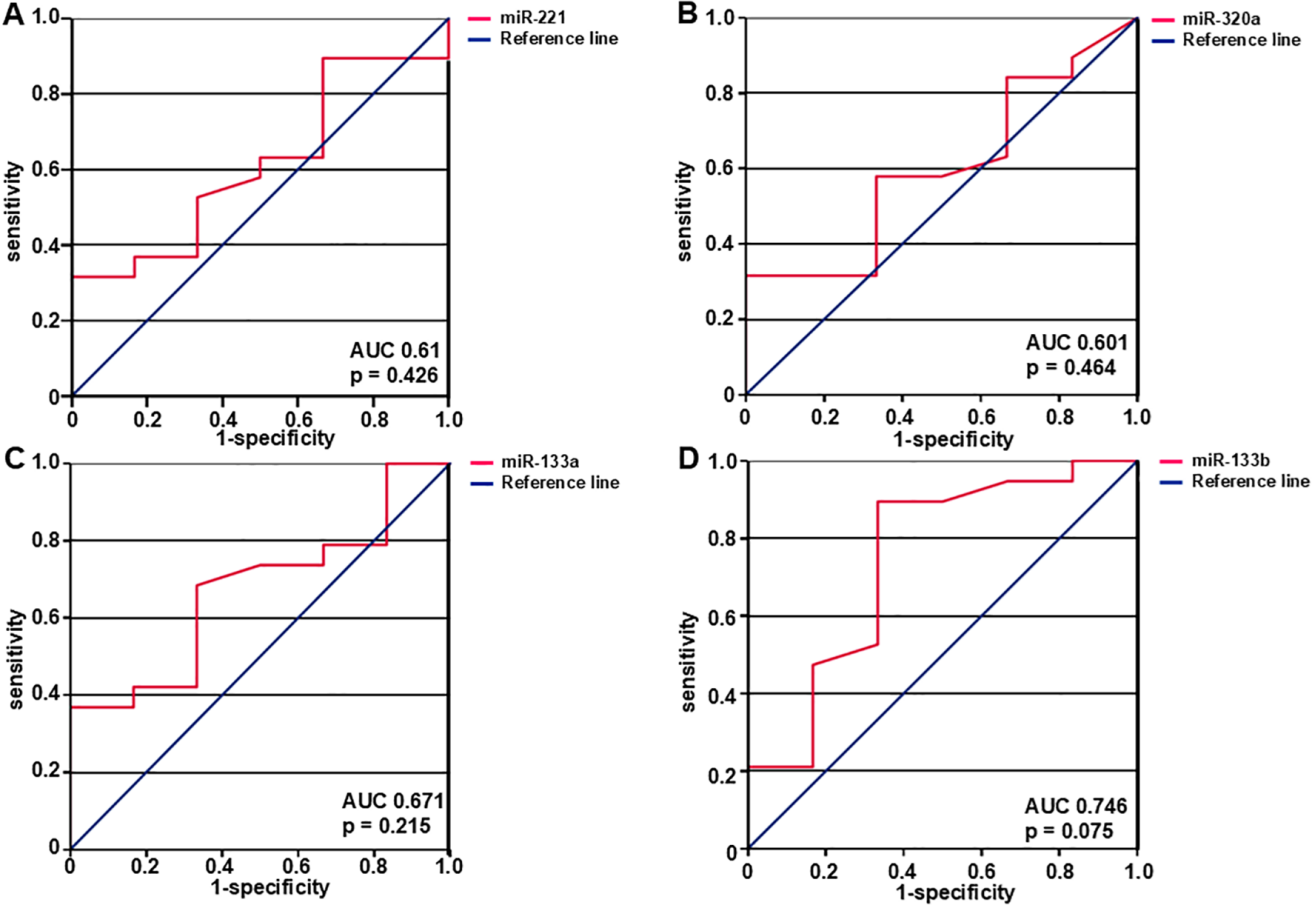
The diagnostic ability of miRNAs to predict metastasis was assessed with the use of ROC curves. AUC- and p-values are shown in the figure. Expression levels of miR-221 **(A)**, miR-320a **(B)**, miR-133a **(C)**, and miR-133b **(D)** predict the likelihood of developing metastasis. MiR-221, miR-320a, miR-133a, and miR-133b expression levels were examined, and the corresponding AUC values were 0.61, 95% confidence interval 0.851 (p = 0.426), 0.601, 95% confidence interval 0.847 (p = 0.464), 0.671, 95% confidence interval 0.901 (p = 0.215), and 0.746, 95% confidence interval 1.0 (p = 0.075) respectively. N = 25 (local and primary tumors).

ROC curve analysis was carried out to assess how effectively the miRNA expression levels predict the likelihood of developing metastases over time at initial presentation of LMS. (**Figure 4**). MiR-221 exhibited an area under the curve (AUC) of 0.61 with 95% confidence interval of 0.851 (p = 0.426) indicating a non-significant level of p (**Figure 4A**). Similarly, miR-320a was evaluated and found to have an AUC of 0.601 with 95% confidence interval of 0.847 (p = 0.464) (**Figure 4B**) and miR-133a had an AUC of 0.671 with 95% confidence interval of 0.901 (p = 0.215) (**Figure 4C**). MiR-133b showed the highest AUC of 0.746 with 95% confidence interval of 1.0 and p = 0.075 (**Figure 4D**).

### 3.4 Correlation of miR-221, miR320a, miR-133a, and miR-133b with clinical and histopathological features

Using Spearman-Rho test miR-221, miR320a, miR-133a, and miR-133b expression (fold change) was correlated to tumor size (T1-T4), grading (G1, G2, and G3), and age (**Table 3**). There was no significant correlation of the four miRNAs to the clinical and histopathological parameters.

**Table 3:** Correlation of miR-221, miR-320a, miR-133a, and miR-133b with clinical and histopathological parameters.

N = 33				
miRNA	miR-221	miR-320a	miR-133a	miR-133b
Grading	r = 0.165 p = 0.358	r = 0.016 p = 0.929	r = 0.186 p = 0.299	r = 0.26 p = 0.144
Tumor size	r = -0.051 p = 0.788	r = 0.119 p = 0.530	r = 0.131 p = 0.492	r = -0.181 p = 0.339
Age	r = -0.011 p = 0.950	r = 0.064 p = 0.725	r = 0.056 p = 0.757	r = -0.014 p = 0.939

*significant p < 0.05.

## 4 Discussion

Leiomyosarcoma (LMS) is characterized by an aggressive nature and poorly understood molecular pathogenesis. As treatment options for metastasized LMS are limited, there is significant clinical interest in understanding the pathogenesis of the disease. Currently, standard clinical and histopathologic characteristics, i.e. tumor size and grading are used to predict prognosis in LMS (51, 52). In sarcoma there are currently no molecular biomarkers in clinical practice. In addition to studies of proteins and their corresponding genes to identify new mechanisms of sarcoma pathobiology, noncoding regions of the genome, coding for miRNAs have come into focus. Several miRNAs have been found to be dysregulated in sarcomas (10, 66). MicroRNAs are small regulatory RNA molecules that modulate the expression of their target mRNAs. MiRNAs have oncogenic or tumor suppressor properties according to the molecular pathways of the targeted mRNAs. A single miRNA can influence a network of different signaling pathways (53).

To identify potential biomarkers in sarcoma, several microarray-based studies have been performed (54, 55). Lee et al., 2016 (56) identified a miRNA based molecular classification in LMS with association to tumor grade. Moreover, miR-221, miR-320a, miR-133a, and miR-133b were described to be upregulated in LMS (16–18, 35, 57). MiR-221 has been found as an oncogenic miRNA in LMS (57) and is significantly upregulated in LMS compared to benign leiomyomas. MiR221 may thus be considered as potential biomarker for distinguishing malignant LMS from benign smooth muscle tumors. Guled at al., 2014 (17) compared miRNA-profiles of LMS and undifferentiated pleomorphic sarcoma (UPS). In LMS five miRNAs, including miR-320a, classified the sarcomas in one UPS group and two LMS groups. Comparing LMS and normal smooth muscle, significant overexpression of miR-133a and miR-133b in LMS was detected (35). Overall, studies of miR-221, miR-320a, miR-133a, and miR-133b in LMS are scarce. With this study we set out to further investigate the role of these miRNAs in LMS.

33 patients with LMS were included. MiR-221, miR-320a, miR-133a, and miR-133b expression was analyzed in tumor and adjacent non-tumor LMS tissue from FFPE sections. Comparing miRNA expression between tumor and adjacent non-tumor LMS tissue we found a significant elevation of miR-221, miR-320a, and miR-133a in tumor tissue. MiR-133b was downregulated. Analysis of these miRNAs may facilitate histopathological diagnosis by distinguishing LMS from other sarcoma types.

To analyze described target mRNAs of miR-221, miR-320a, miR-133a, and miR-133b we chose *CDKN1B-, TGFR1-*, and *IGFR1* mRNA. Galardi et al., 2007 (58) found an inverse relationship between increased miR-221 expression and control of cell cycle progression. The cyclin-dependent kinase inhibitors P27kip1 and P57kip2, which are significant regulators of cell cycle progression, are downregulated on miRNA-221 overexpression, promoting cell proliferation (58–60). We detected no significant difference between *CDKN1B* mRNA expression in tumor versus non-tumor tissue. As target mRNA for miR-320a we analyzed *TGFBR1* mRNA. In endometrial carcinoma, miR-320a is downregulated, and its mimic prevents endometrial cancer cells from migrating and invading by specifically targeting eIF4E. Upon eIF4E elevation in endometrial carcinoma, TGFBR1 induced HEC-1A cells to undergo endothelial-to-mesenchymal transition (EMT). A significant factor controlling the EMT process was TGFBR1 (61). In our study no significant change of *TGFBR1* was detected. Multiple lines of evidence suggest that the upregulation of insulin-like growth factor 1 receptor (IGF1R) plays a crucial role in promoting carcinogenesis and drug resistance in gastric cancer. In many human solid malignancies, IGF1R expression was increased with an association to poor outcome (62–64). By targeting *IGF1R* and inhibiting the downstream AKT and ERK signal pathway, miR-133a suppresses cell proliferation, induces cell cycle arrest at the G0/G1 stage, and promotes apoptosis in osteosarcoma, hepatocellular carcinoma, and gastric cancer (47, 48), suggesting an inverse correlation of miR-133a and IGF1R. Our study in LMS showed no significant change in *IGF1R* mRNA expression. Since many miRNAs change target translation without influencing target mRNA levels, an alteration of *CDKN1B-, TGFBR1-* or *IGF1R* mRNA is not excluded. Due to the control of complete signaling networks small changes in mRNA that we did not detect, may be amplified.

Since metastasis formation is the major threat in sarcomas, we sought to identify miRNAs predictive for metastatic risk. Such biomarkers for metastasis would allow tailoring of adjuvant chemo- or radiation therapy and higher surveillance for early detection of metastasis. To adequately chose neoadjuvant- or adjuvant treatment at initial LMS presentation and to reduce risk for metastasis, biomarkers are urgently needed. We therefore analyzed miR-221, miR-320a, miR-133a, and miR-133b in local tumors, primary tumors, and metastases. Local tumors did not metastasize, whereas primary tumors were metastasized at the time of initial presentation or later during the clinical course. MiR-221, miR-320a, miR-133a, and miR-133b were not significantly upregulated in primary tumors compared to local tumors, local tumors compared to metastases or in metastatic tissue versus primary tumors. The ROC-curves revealed no significant prediction of metastasis for miR-221, miR-320a, miR-133a, and miR-133b. P = 0.075 for the ROC-curve for miR-133b may indicate a possibility to reach significance for miR-133b with higher sample numbers.

There was no significant correlation of miR-221, miR-320a, miR-133a, and miR-133b with clinical or histopathological parameters as tumor size, grading, or age. Several studies analyzed the metastatic pattern of LMS. Tigchelaar et al., 2022 (3) found 23.3% of LMS with metastases at initial presentation and 68.5% of metastases at later time points, demonstrating metastatic disease in 91.8% of LMS patients. In primary retroperitoneal LMS a lower number of local relapse after tumor resection, but a high rate of metastases compared to retroperitoneal liposarcoma was detected (65). These findings may explain our results comparing miR-221-, miR-320a-, miR-133a-, and miR-133b expression in local tumors to primary tumors and metastases with no significant difference, indicating, that LMS has a high metastatic potential at initial presentation with no change in miRNA-expression between non-metastasizing and metastasizing tumors in several miRNAs.

In conclusion, our results demonstrate that miR-221, miR-320a, and miR-133a are significantly upregulated in LMS tumor tissue as compared to adjacent non-tumor tissue. Besides the possible use in histopathological diagnosis these miRNAs are candidates for evaluation as biomarkers in the plasma auf LMS-patients, potentially allowing early and simple diagnosis at initial presentation of LMS and recurrent leiomyosarcoma. The target mRNAs *CDKN1B, TGFR1,* and *IGF1R* showed no significant change between tumor and non-tumor tissue. Comparing local tumors, primary tumors and metastases in LMS patients, no significant change in miR-221-, miR-320a-, miR-133a-, and miR-133b expression in local tumors compared to metastases as well as in local tumors compared to primary tumors or primary tumors compared to metastases was found. ROC curves of miR-221, miR-320a, and miR-133a did not predict metastasis, Expression difference between primary and local tumors as well as the ROC curve of miR-133b may reach significance in the prediction of metastasis with higher sample numbers.

As sarcomas are rare tumors our patient cohort was small but homogenous, since we only included LMS tissue samples and no other sarcoma types. The relatively small subgroup sample sizes result in diminished statistical power. This study is exploratory and requires validation by an independent cohort. Further limitations of this study are the sample origin from different organs and the challenging isolation of miRNAs from archival formalin-fixed paraffin-embedded (FFPE) samples. Utilizing larger sample sizes and advanced technologies such as laser microdissection may result in more robust data. Future work, such as validating miRNA-mRNA interactions using functional assays e.g., luciferase reporter assays or knockdown/overexpression studies, expression patterns in circulating biofluids, or confirming findings in larger, multi-institutional cohorts may expand the data.

Further analysis of these candidate miRNAs in a larger patient cohort is required and may lead to the establishment of miR-221, miR-320a, miR-133a, and miR-133b into routine use as prognostic biomarkers for the diagnostic workup of leiomyosarcomas. To warrant these findings, expression analyses using more LMS samples are needed in prospective future studies. Several results in this study were borderline significant, thus a higher sample number may lead to reach significance. Our work provides an important step in the complicated search to understand the molecular mechanisms of sarcomas, hopefully leading to continuation of further investigations and better treatment options in this dismal disease.

## Data Availability

The raw data supporting the conclusions of this article will be made available by the authors, without undue reservation.
