# Supplementary material for: MiR-221, miR-320a, miR133a, and miR-133b as potential biomarkers in leiomyosarcoma
Source: Front Oncol. 2025 Jun 20;15:1577859. doi: 10.3389/fonc.2025.1577859 (PMC12236100; doi:10.3389/fonc.2025.1577859)
Supplement: Supplementary file 1 [file Table1.docx]

**Supplementary tables**

**Suppl. Table 1**

**Studied patients (N = 33) and characteristics**

| Variables | Case | N (%) |
| --- | --- | --- |
|  |  |  |
| Gender | Male | 4 (12) |
|  | Female | 29 (88) |
|  |  |  |
| Tumor stage | Local | 6 (18) |
|  | Primary | 19 (58) |
| Tumor size (T) | Metastasis  T1  T2  T3  T4 | 8 (24)  14 (43)  10 (30)  3 (9)  6 (18) |
|  |  |  |
| Age (years) | Range | 40-78 |
|  | Median | 60 |
|  |  |  |
| Grading | G1 | 3 (9) |
|  | G2 | 18 (54) |
|  | G3 | 12 (36) |
|  |  |  |
| Primary organ involved | Uterus | 6 (18) |
|  | Leg | 5 (15) |
|  | GI-tract | 5 (15) |
|  | Liver | 2 (6) |
|  | Heart | 1 (3) |
|  | Lung  Vena cava  Pelvis/retroperitoneum  Mediastinal | 2 (6)  3 (9)  8 (24)  1 (3) |
|  |  |  |
| Time to metastasis | No metastasis | 6 (18) |
|  | Metastasis (median, range) | 27 (82)  Range = 0 - 72 months  Median = 12 months |

**Suppl. Table 2**

**Presence of miR-221, miR-320a, miR-133a, and miR-133b in different cell lines**

| Cell lines | miR-221 | miR-320a | miR-133a | miR-133b |
| --- | --- | --- | --- | --- |
| FaDu | 0 | 1 | 1 | 0 |
| Lung tissue | 0 | 1 | 0 | 1 |
| HCC78 | 0 | 1 | 1 | 0 |
| HepG2 | 1 | 1 | 0 | 0 |
| SkBr-3 | 0 | 1 | 0 | 1 |

miR positiv (1), miR negativ (0)

**Suppl. Table 3**

**Primer sequence of target genes**

| Target (product size) | Sequence (5´-3´) |
| --- | --- |
| *TGFBR1*(354 bp) |  |
| Forward | ATCCCAAACAGATGGCAGAG |
| Reverse | CGCAACTCAGTCAACAGGAA |
| *IGF1R* (438 bp) |  |
| Forward | ACGCCAATAAGTTCGTCCAC |
| Reverse | CAGGCTCCATCTCCTCTTTG |
| *CDKN1B* (483 bp) |  |
| Forward | ACCTGCAACCGACGATTCTT |
| Reverse | CTCTTCATACCCCGCTCCAC |
| *RPL37A* (190 bp) |  |
| Forward | CTCCGGAAAATGGTGAAGAA |
| Reverse | TTCTGATGGCGGACTTTACC |
